# Safeguarding the right to die

**DOI:** 10.1016/j.eclinm.2025.103223

**Published:** 2025-04-22

**Authors:** eClinicalMedicine

In November, 2024, backbench Labour Member of UK Parliament (MP) Kim Leadbeater introduced the Terminally Ill Adults (End of Life) Bill, which aims to legalise assisted dying in England and Wales for terminally ill patients with a life expectancy of less than 6 months, to the UK's House of Commons. The bill was supported by 330 MPs (and rejected by 275) and will undergo extensive review and further votes in the Houses of Commons and Lords before it becomes law. The bill is due to reach the report stage towards the end of April, 2025, in which MPs will debate further amendments. However, other members of the British Isles are further ahead; the Isle of Man, a self-governing British Crown Dependency, became the first of the British Isles to legalise assisted dying for terminally ill people with fewer than 12 months to live in March, this year. Additionally, Scotland introduced a bill in March, 2024, and it is in early stages of consideration. Many stages remain before the Terminally Ill Adults (End of Life) Bill becomes law, but if or when it does, England and Wales will join a growing list of countries in which the right to die is enshrined in law.

Around the world, more than 300 million people now live in countries where assisted dying is legal, including Switzerland, the first country to make assisted dying legal in 1942, the Netherlands and Belgium, where assisted dying is legal for people with incurable illnesses that cause unbearable suffering, and Austria and Spain, who have legalised assisted dying for people with terminal illnesses or unbearable suffering. Elsewhere in Europe, France is progressing with its own legislation, after dividing it into two bills that focus on palliative care and on “aid in dying” in early April.

In the USA, assisted dying is legal in ten states, and Oregon was the first state to legalise it in 1997. Since its introduction in Oregon, assisted dying has remained limited to the terminally ill. In Canda, assisted dying was introduced for the terminally ill in 2016, but the law was amended in 2021 to extend to people with irreversible illnesses or disabilities.

In the UK, those who oppose assisted dying often refer to the so-called slippery slope, where they believe the right to die could be extended to more groups, like in Canada, and ensuing changes in attitudes might result in abuse and put the vulnerable at risk. Indeed, in Canada, there have been reports of people that have asked for assistance on the basis of other factors, including an “unmet social need”. Additionally, they suggest that legalising assisted dying could undermine trust in doctors, with individuals seeing themselves as a burden, and that the role of the doctor is to extend life. They also argue that assisted dying would be unnecessary if effective palliative care was available and could impede its pursuit.

Those on the other side of the UK debate suggest that the slippery slope is not an inevitability, such as in Oregon, which has steadfastly limited assisted dying to the terminally ill. They state that the UK already has guidance and safeguards for end-of-life practices, such as withdrawal of life-sustaining care, that protect the vulnerable and that they can be extended to assisted dying to prevent abuse. They also argue that physicians should be able to provide compassionate relief and support suffering patients and should not impose their own beliefs regarding the sanctity of life on the patient (legislation could also contain conscientious objector clauses), and patients should be offered autonomy and dignity in death. Finally, there is the argument that, no matter how good palliative care becomes, there will always be suffering so great that it cannot be relieved.

Despite the arguments on both sides, public opinion is in favour of assisted dying, with a study from October, 2024, suggesting that 63% of people in England and Wales support legislation within the next 5 years. However, 61%, including 53% of supporters, were worried about people being pressured or coerced into an assisted death, and 48% of supporters would rethink their position if they felt that an assisted death was motivated by lack of access to appropriate care. Indeed, in addition to issues affecting health care in general, palliative care in the UK is facing a crisis; hospices lack funding and are closing, more than a fifth of patients who require palliative care die without receiving it, and half of families are unhappy with the care their loved ones received.

If bills that legalise the right to die are to proceed and succeed, robust safeguards and clear and well defined eligibility criteria will need to be implemented to eliminate abuse and reassure people that abuse is not possible. The Terminally Ill Adults (End of Life) Bill suggests that individuals will be required to have necessary mental capacity, receive eligibility assessments from two independent doctors, and have expressed a clear and informed wish in two witnessed and signed declarations free from coercion or pressure. Despite these steps, efforts still need to be made to address issues with funding for and access to care, particularly palliative care, if assisted dying is to remain free from abuse and acceptable to the general public.

